# Synthesis and Characterization of 5-Nitro-2-nitratomethyl-1,2,3,4-tetrazole: A High Nitrogen Energetic Compound with Good Oxygen Balance

**DOI:** 10.3390/molecules17055040

**Published:** 2012-05-03

**Authors:** Yuchuan Li, Wei Liu, Siping Pang

**Affiliations:** 1 School of Material Science & Engineering, State Key Laboratory of Explosion Science and Technology, Beijing Institute of Technology, Beijing 100081, China; Email: yuchuanyh@yahoo.com.cn (Y.L.); liuwei1986825@sina.com (W.L.); 2 School of Life Science, Beijing Institute of Technology, Beijing 100081, China

**Keywords:** synthesis, characterization, 5-nitro-2-nitratomethyl-1,2,3,4-tetrazole, crystal structure, quantum calculation, high nitrogen energetic compound, oxygen balance

## Abstract

The synthesis of 5-nitro-2-nitratomethyl-1,2,3,4-tetrazole (**4**) and its full characterization are given here. Compound **4** was synthesized through the nitration of 5-nitro-2-hydroxymethyl-tetrazole (**3**) with fuming nitric acid and acetic anhydride and its structure was characterized by MS, FT-IR, ^1^H-NMR and ^13^C-NMR techniques. The crystal structure of **4** was determined by X-ray single crystal diffraction analysis. The compound belongs to the orthorhombic system with space group *P*na2(1), and its crystal parameters were *a =* 2.121(8) nm, *b =* 0.5281(19) nm, *c =* 0.6246(2) nm, *Z =* 4, *V* = 0.6995(4) nm^3^, *D*c = 1.805 g/cm^3^, *F*(000) = 384, *μ* = 0.174 mm^−1^. A theoretical study of **4** has been performed, using quantum computational density functional theory (B3LYP methods) with 6-31G* basis sets as implemented in the Gaussian 03 program suite. The obtained heat of formation (HOF) for **4** was 228.07 kJ·mol^−1^, the detonation pressure (*P*) values calculated for **4** was 37.92 GPa, the detonation velocity (*D*) can reach 9260 m·s^−1^, and the oxygen balance was zero (Q), making **4** a competitive energetic compound.

## 1. Introduction

Azoles which have nitrogen-containing five-membered heterocycles are traditional sources of energetic materials, since the N–N bonds in the ring are stabilized by pseudoaromatic electron delocalization and are relatively insensitive. Tetrazoles are a typical high-nitrogen compound with high nitrogen content (80%), that possess a high positive heat of formation, and the tetrazole ring system is a powerful building block for high-energy compounds. Other than the high heats of formation, for a compound to be a high-performing energetic, a high oxygen balance is required. Oxygen balance is an expression that is used to indicate the degree to which an explosive can be oxidized and is easily calculated by the equation Ω (%) = (*w* − 2*x* − 1/2*y* − 2*z*) 1600/M (*w*: number of oxygen atoms, *x*: number of carbon atoms, *y*: number of hydrogen atoms, *z*: number of sulfur atoms). Therefore, many tetrazole derivatives have been studied extensively as unique energetic compounds [1,2]. Moreover, new approaches, including ionization of tetrazole compounds, introduction of nitro or azo groups, and *N*-oxidation of nitrogen in heterocycles have been developed to improve the properties of tetrazole derivatives [3,4,5]. These suggestions indicate that the combination of high-nitrogen heterocycles with different oxygen-containing groups (such as nitro group, *N*-oxidation, *etc*.) is an effective method to improve the properties of tetrazole energetic compounds, especially on the aspects of enhancing oxygen balance and increasing density [4,6,7,8]. 

Whereas the chemistry and application of 5-nitrotetrazole salts as energetic materials has been studied extremely [7,8], the corresponding chemistry of 5-nitrotetrazole neutral molecule derivatives has only recently been the subject of systematic investigations [9,10,11]. There is a pressing need in the energetic materials field to be able to incorporate more oxygen atoms onto derivatives of the already high-performing tetrazole ring. Introducing nitrate ester groups onto the tetrazole ring may prove to be a viable solution to this challenge and may push the limits of well-explored tetrazole chemistry into a new, unexplored, dimension. Nitrate esters are one of the oldest classes of energetic materials which display easy syntheses, high oxygen content and good combustion characteristics. The 2-substituted 5-nitrotetrazoles, especially those with oxygen-containing group substituents, have good oxygen balance and high density [10,11,12,13]. To the best of our knowledge, although the synthesis of 5-nitro-2-nitratomethyl-1,2,3,4-tetrazole (5-nitro-1,2,3,4-tetrazole-2-ylmethyl nitrate, **4**) has been performed in the past [10], no physical properties or proof of structure were given for the compound. For the past several years we have been working on the development of tetrazole-base energetic materials [2,14,15], as part of our continued efforts in the field of high-nitrogen energetic compounds. The improved synthesis and characterization of compound **4**, together with a single crystal X-ray structure determination and the quantum chemistry calculation are now reported in this paper.

## 2. Results and Discussion

### 2.1. Synthesis and Spectral Studies of 5-Nitro-2-nitratomethyl-1*,*2*,*3*,*4-tetrazole *(**4**)*

**Scheme 1:**
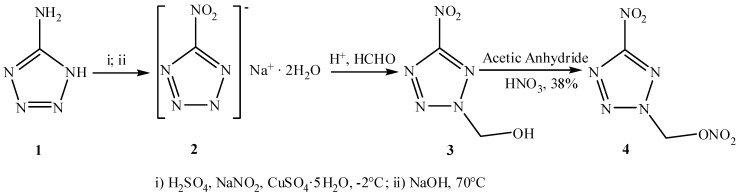
Synthesis of 5-nitro-2-nitratomethyl-1,2,3,4-tetrazole (**4**).

The compound **4** was synthesized in moderate yield (38%) by nitration with dilute nitric acid and acetic anhydride of the alcohol **3**, which was prepared from the previously described 5-nitrotetrazole sodium salt (**2**) by reaction with formaldehyde in the presence of a strong acid (such as H_2_SO_4_, *etc*.) followed by work up by diethyl ether extraction (Scheme 1), using some improvements made by the authors [2,10,16], in particular the reaction time was shortened and the operation was made more convenient. It was also noteworthy that the crude product thus obtained was pure enough for further use. Compound **3** was also prepared from 5-aminotetrazole by a one-pot method, the only difference between the two methods being the color of the target product: the one produced by the one-pot method appears little yellow and the other is white. 

Compound **4** was characterized through spectroscopic data. The IR spectrum of **4** shows the characteristic single –ONO_2_ peak at 1,676 cm^−1^ and a sharp C–NO_2_ peak at 1,562 cm^−1^, while the skeletal vibrations of the azole ring appear at 1,491, 1,421 and 1,385 cm^−1^. The ^1^H-NMR spectrum shows a characteristic C–H singlet at δ 7.30 ppm, which shows a significant downfield shift due to the strong negative inductive effect of the tetrazole ring and the nitro group. In the ^13^C-NMR spectrum, a characteristic C–NO_2_ peak appears at δ 168.90 ppm and C–ONO_2_ appears at δ 78.31 ppm, with both appearing at uncharacteristic low field compared to their analogues. Although we failed to observe a molecular ion peak in the mass spectrum, peaks for nitratomethyl anion (75.8), 5-nitro-2-methene-1,2,3,4-tetrazole anion (127.1) and 5-nitro-1,2,3,4-tetrazole anion (113.8, 100%) were observed. 

### 2.2. Crystal Structure Determination

**Figure 1:**
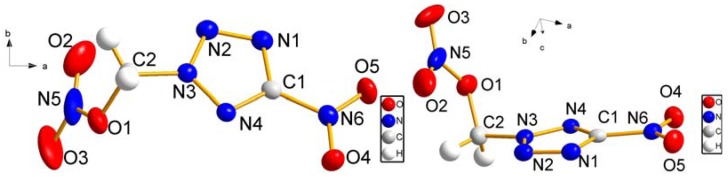
Molecular structure of **4** at 50% probability ellipsoids.

**Figure 2:**
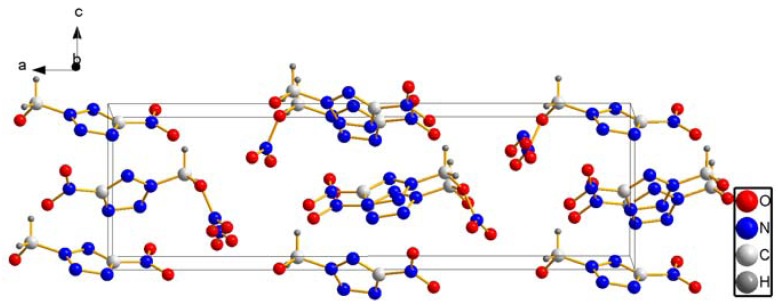
The packing diagram of compound **4**.

The crystal structure of **4** adopts a stereostructure whose non-hydrogen geometries are summarized, along with bond lengths and angles which are in the usual ranges, in Table 1 and Table 2, Figure 1 and Figure 2. The −NO_2_ and −CH_2_− almost lie in the plane of the tetrazole ring to which they are attached, whereas the –ONO_2_ is twisted out of the plane (torsion angles: N(5)−O(1)−C(2)−N(3), −85.49(15)°, N(4)−N(3)−C(2)−O(1), −68.73(18)°, N(2)−N(3)−C(2)−O(1), 114.84(15)°). Moreover, it is worth noting that the interatomic distances in compound **4** vary considerably, from 1.193(2) Å [O(3)−N(5)] to 1.4595(18) Å [N(3)−C(2)]. N(3)−C(2) is the longest bong length [1.4595(18) Å], which might be the easiest chemical bond to break down, giving the nitratomethyl anion (*m/z* 75.8) and 5-nitro-1,2,3,4-tetrazole anion (*m/z* 113.8, 100%) correspondingly. 

**Table 1:** Crystal data and refinement for 5-nitro-2-nitratomethyl-1,2,3,4-tetrazole (**4**).

Empirical formula	C_2_H_2_N_6_O_5_
Formula weight	190.10 g·mol^−1^
Temperature	148(2) K
Wavelength	0.71073 Ǻ
Crystal system	Orthorhombic
Space group	Pna2(1)
Unit cell dimensions	a = 2.1209(8) nm *α* = 90°
	b = 0.52805(19) nm *β* = 90°
	c = 0.6246(2) nm *γ* = 90°
Volume	699.5(4) nm ^3^
Z	4
Density calculated	1.805 g·cm^−3^
Absorption coefficient	0.174 mm^−1^
F(000)	384
Crystal size	0.54 × 0.52 × 0.08 mm^3^
Theta range for data collection	3.262° to 29.103°
Limiting indices	−25 ≤ h ≤ 29, −7 ≤ k ≤ 7, −8 ≤ l ≤ 7
Reflections collected / unique	5552 / 1749 [R(int) = 0.0263]
Completeness to theta = 29.13	99.8%
Refinement method	Full-matrix least-squares on F^2^
Goodness-of-fit on F^2^	0.998
Final R indices (I > 2sigma(I))	R1 = 0.0296, wR_2_ = 0.0594
R indices (all data)	R1 = 0.0333 wR_2_ = 0.0609
Largest diff. peak and hole	0.205 and −0.133 e.A^−3^
CCDC No.	868752

**Table 2:** Interatomic distances (Å) and angles (°) for compound **4**.

Atoms	Parameter	Atoms	Parameter
**Distances**			
O(1)–C(2)	1.407(19)	N(1)–C(1)	1.338(18)
O(1)–N(5)	1.448(2)	N(2)–N(3)	1.333(17)
O(2)–N(5)	1.198(2)	N(3)–N(4)	1.324(16)
O(3)–N(5)	1.193(2)	N(3)–C(2)	1.460(18)
O(4)–N(6)	1.226(16)	N(4)–C(1)	1.316(17)
O(5)–N(6)	1.222(16)	N(6)–C(1)	1.446(19)
N(1)–N(2)	1.310(17)		
**Angles**			
C(2)–O(1)–N(5)	113.2(14)	O(2)–N(5)–O(1)	117.6(15)
N(2)–N(1)–C(1)	104.7(12)	O(5)–N(6)–O(4)	125.6(12)
N(1)–N(2)–N(3)	105.9(11)	O(5)–N(6)–C(1)	117.0(12)
N(4)–N(3)–N(2)	114.6(11)	O(4)–N(6)–C(1)	117.4(11)
N(4)–N(3)–C(2)	123.4(12)	N(4)–C(1)–N(1)	115.4(13)
N (2)–N(3)–C(2)	122.0(12)	N(4)–C(1)–N(6)	122.8(13)
C(1)–N(4)–N(3)	99.5(12)	N(1)–C(1)–N(6)	121.8(12)
O(3)–N(5)–O(2)	132.0(2)	O(1)–C(2)–N(3)	110.6(12)
O(3)–N(5)–O(1)	110.4(19)		
**Torsion**			
C(1)–N(1)–N(2)–N(3)	0.29(14)	N(2)–N(1)–C(1)–N(4)	−0.40(17)
N(1)–N(2)–N(3)–N(4)	−0.12(16)	N(2)–N(1)–C(1)–N(6)	−178.11(12)
N(1)–N(2)–N(3)–C(2)	176.60(12)	O(5)–N(6)–C(1)–N(4)	−179.64(13)
N(2)–N(3)–N(4)–C(1)	−0.10(15)	O(4)–N(6)–C(1)–N(4)	1.10(2)
C(2)–N(3)–N(4)–C(1)	−176.78(13)	O(5)–N(6)–C(1)–N(1)	−2.10(2)
C(2)–O(1)–N(5)–O(3)	177.24(15)	O(4)–N(6)–C(1)–N(1)	178.60(13)
C(2)–O(1)–N(5)–O(2)	−2.20(2)	N(5)–O(1)–C(2)–N(3)	−85.49(15)
N(3)–N(4)–C(1)–N(1)	0.31(16)	N(4)–N(3)–C(2)–O(1)	−68.73(18)
N(3)–N(4)–C(1)–N(6)	177.99(12)	N(2)–N(3)–C(2)–O(1)	114.84(15)

### 2.3. Differential Scanning Calorimety (DSC)

DSC measurement to determine the thermal behavior of compound **4** was carried out with a heating rate of 5 °C·min^−1^, using dry oxygen-free nitrogen as atmosphere with a flowing rate of 20 mL·min^−1^(1.5000 mg of powder). An exothermic peak of compound **4** was observed at 170.81 °C, which indicates a very high decomposition temperature.

### 2.4. Quantum chemistry Calculation

The crystal density of **4** was calculated to be 1.801 g·cm^−3^ in good agreement with experimental one (1.805 g·cm^−3^), and the heat of formation of **4** was predicted to be +228.07 kJ·mol^−1^ (+1,200 kJ·kg^−1^) by well established methods (Table 3). Compound **4** has zero oxygen balance, high density and heat of formation. The detonation pressure (*P*) values calculated for **4** was 37.92 GPa, and the detonation velocity (*D*) can reach 9,260 m·s^−1^, making **4** a competitive energetic material (Table 3). 

**Table 3:** The comparison of some properties of compound **4**, TNT, RDX and HMX [17].

Compound	HOF(kJ·mol^−1^)	*ρ*(g·cm^−3^)	*D*(km·s^−1^)	*P*(GPa)	*Q*(%, CO_2_)
**4**	+228.07	1.801	9.26	37.92	0
**TNT**	−52.22	1.650	7.02	20.70	−73.9
**RDX**	−192	1.816	8.75	34.7	−21.6
**HMX**	−250	1.90	9.09	39.0	−21.6

### 2.5. Impact Sensitivity Test (H_50_)

The impact sensitivity of **4** was tested on a type 12 tooling according to ‘up and down’ method, a 2.5 kg weight was dropped from a set height onto a 20 mg sample of an explosive placed on 150 grit garnet sandpaper. Each subsequent test was made at the next lower height if explosion occurred and at the next higher height if no explosion happened. Fifty drops were made from different heights and an explosion or nonexplosion was recorded, the test result (H_50_) of **4** is 16.6 cm (4.1 J) [18]. Test conditions: 26 °C (temperature), 47% (relative humidity).

## 3. Experimental

***Caution***: Although we have not experienced any problems during the synthesis of the reported compounds, standard safety precautions (leather gloves, face shield and ear plugs) should be taken when handling this or any suspected energetic material.

### 3.1. General Methods

All materials were commercially available and used as received. The reactions were monitored by thin-layer chromatography (TLC), and the compounds were detected by examination under ultraviolet light. Melting point was determined using XT4 microscope melting point apparatus and are uncorrected. FT-IR spectra were recorded using KBr pellets for solids on a Bruker Tensor 27 spectrometer. ^1^H- and ^13^C-NMR spectra were recorded on a Bruker AM-400 MHz spectrometer (Zurich, Switzerland) operating at 400 MHz and 100 MHz, respectively, with CDCl_3_ as the locking solvent. Chemical shifts are reported in ppm relative to TMS. Electrospray ionization mass spectrum (ESI-MS) was performed on a Bruker APEXII FT-ICR MS (Billerica, MA, USA). Elemental analyses were performed on Elementar Vario EL (Bremen, Germany). DSC studies were carried out on a TA-DSC Q2000 (New Castle, DE, USA). Detonation pressure and velocity were predicted by the Kamlet-Jacobs equations. Computations were performed by using the Gaussian 03 (Revision F.02) suites of programs [19]. The B3LYP/6-31G** method was used to calculate the molecular volumes for the energetic triazolium salts. The volume of each ion was deﬁned as inside a contour of 0.001 electrons/bohr^3^ density that was evaluated using a Monte Carlo integration.

### 3.2. Synthesis of 5-Nitro-2-nitratomethyl-1*,*2*,*3*,*4-tetrazole *(**4**)*

5-Nitro-2-nitratomethyl-1,2,3,4-tetrazole (**4**) was synthesized by a modified literature procedure [2,5,10,15] as follows: copper sulfate pentahydrate (11.00 g, 44 mmol) and sodium nitrite (20.80 g, 300 mmol) were loaded into a 500 mL plastic beaker and dissolved in distilled water (100 mL). The mixture was cooled to −2–3 °C. Meanwhile, a solution of compound **1** (8.626 g, 101 mmol) and copper sulfate pentahydrate (0.40 g, 1.6 mmol) in water (150 mL) and concentrated sulfuric acid (10 g, 98%) was prepared, and added to the previous solution dropwise over 20 min by means of a peristaltic pump. The suspension was stirred at room temperature for another 20 min and reacted with sodium hydroxide (8.00 g, 200 mmol) for 1 h in a water bath at 68–70 °C under vigorous stirring. The black-brown copper(II) oxide, a by-product, was filtered hot through Celite and the Celite was subsequently washed with 50 × 2 mL hot water, yielding a pale yellow solution. Concentrated sulfuric acid (10 g, 98%) was added to the solution and stirred for 10 min. The mixture was extracted three times with dichloromethane (450 mL) and *tri*-*n*-butylamine (15 g). A sodium hydroxide solution (50%, 6.5 g) was added to the organic phase and stirred for 30 min. The precipitate was filtered, dried naturally, and then recrystalized from acetone/dichloromethane to afford a good yield (13.0 g, 69%) of compound **2**. 

Compound **2** (13.0 g, 75 mmol) was dissolved in dilute sulfuric acid (140 mL, 7%). An aqueous solution of formaldehyde (37–40%, 19 mL) were added with stirring at 5–10 °C. The mixture was kept for 24 h at about 18°C and extracted with ether (90 mL × 3), and the extract was dried over magnesium sulfate and evaporated under reduced pressure to obtain 6.1 g (68%) of compound **3** as a colorless crystalline substance. The alcohol **3** (6.1 g, 42 mmol) was slowly added with stirring at 5 °C to a nitrating mixture prepared from acetic anhydride (60 mL) and fuming nitric acid (95–98%, 60 mL) at 0–5 °C. The reaction mixture was stirred for 3 h at 10 °C and poured onto crushed ice (300 g), then the precipitate was filtered off and washed with ice water (80 mL). The crude product was recrystalized from ether and a moderate yield (3.8 g, 38%) of **4** as a white powder was obtained: m.p. 72.7–74.0 °C (lit. [10] 74–75 °C); FT-IR spectrum (KBr, cm^−1^, vs: very strong; s: strong; m: medium; w: weak): 631, 815, 983, 1049 (C–O, m), 1178, 1284 (O–NO_2_, vs), 1346 (C–NO_2_, m), 1491, 1421, 1385, 1562 (C–NO_2_, vs), 1676 (O–NO_2_, vs); ^1^H-NMR (CDCl_3_): *δ* 7.30 (s, 2H, CH_2_); ^13^C-NMR (CDCl_3_): *δ* 78.31 (–CH_2_–), 167.49; MS (ESI) *m/z*: 127.1 [M–ONO_2_]^+^, 113.8 [M–CH_2_ONO_2_]^+^, 75.8 [CH_2_ONO_2_]^+^. 

### 3.3. X-Ray Data Collection and Structure Refinement

Single crystals of **4** suitable for X-ray crystallographic analysis were obtained by slow recrystallization from a diethyl ether solution of **4** at room temperature. Single crystals of compound **4** were mounted on Rigaku RAXIS RAPID IP diffractometer equipped with a graphite-monochromatized MoKα radiation (λ = 0.71073 Å). Data were collected by *ω* scan technique. The structure was solved by direct methods with SHELXS-97 and expanded by using the Fourier technique. The non-hydrogen atoms were refined anisotropically. The hydrogen atom was determined with theoretical calculations and refined with an isotropic vibrational factor. For additional data please see its CIF files.

### 3.4. Calculation Details

**Scheme 2:**
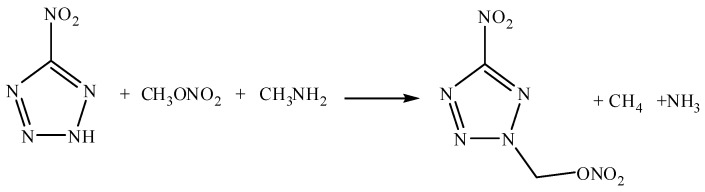
Isodesmic reaction for5-nitro-2-nitratomethyl-1,2,3,4-tetrazole (**4**).

Computations were performed using the Gaussian03 suite of programs [20]. The elementary geometric optimization and the frequency analysis are carried out at the level of Becke three Lee-Yan-Parr (B3LYP) Functionals with 6-31G(d) basis set [21,22,23]. All of the optimized structures were characterized to be local energy minima on the potential surface without any imaginary frequencies. The gas phase heat of formation (HOF) of compound **4** was determined by using the isodesmic reactions method [24]. The isodesmic reactions were carried out as described by Hakima Abou-Rachid (Scheme 2) [23]. 

**Figure 3:**
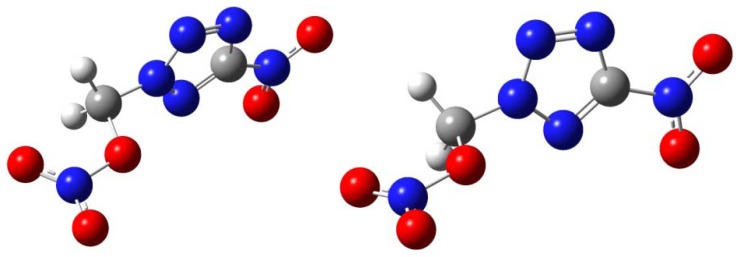
The optimized structure of **4** at B3LYP/6-31G(d) level.

The theoretical density (ρ) of compound **4** was obtained from average mole volume indirectly by Monte Carlo method at B3LYP/6-31G(d) level, based on the optimized structures mentioned above. The average molar volume V estimated by Monte-Carlo method based on 0.001 electrons/bohr^3^ density space was the statistical average of 100 volume calculations, then the theoretical density was obtained by ρ = M/V, where M is the molecular weight [25]. The elementary geometric optimization and the frequency analysis could also be carried out at the level of Becke three Lee-Yan-Parr (B3LYP) Functionals with AUG-cc-pVDZ basis set. The optimizd structure is shown in Figure 3, and the cartesian coordinates of this optimized structure are listed in Table 4.

**Table 4:** Cartesian coordinates of the optimized structure **4**.

	X	Y	Z
**C**	0.183381	−0.020822	0.059801
N	−0.153487	−0.039073	1.448934
O	0.540248	0.596306	2.232762
O	−1.162338	−0.708725	1.711098
C	1.733866	1.089352	−2.791983
H	1.823865	0.462761	−3.687781
H	2.716947	1.381967	−2.404333
O	0.929433	2.262760	−3.076652
**N**	1.487473	3.081719	−4.109374
O	2.529516	2.704601	−4.596656
O	0.820637	4.047085	−4.341554
N	−0.474628	−0.813272	−0.866547
N	−0.004918	−0.478340	−2.132613
N	1.131773	0.679581	−0.487432
N	1.036714	0.356866	−1.785979

## 4. Conclusions

An improved procedure for the synthesis of 5-nitro-2-nitratomethyl-1,2,3,4-tetrazole (**4**) is introduced and its structure was characterized by MS, FT-IR, ^1^H-NMR and ^13^C-NMR techniques. The crystal structure of **4** was determined by X-ray single crystal diffraction analysis. A combination of experimental data and theoretical study shows that **4** has high performances (high density and heat of formation, good oxygen balance), which might be of interest for future application as environmentally friendly and high-performing high-nitrogen compound.

## Supporting Information Available

CCDC-868752 contains the supplementary crystallographic data for this paper (excluding structure factor amplitudes). These data can be obtained free of charge via http://www.ccdc.cam.ac.uk/conts/retrieving.html (or from the CCDC, 12 Union Road, Cambridge CB2 1EZ, UK; Fax: +44-122-333-6033; Email:deposit@ccdc.cam.ac.uk).
